# Sleep and Working Memory: Short-Term Links in Daily Life and Long-Term Associations

**DOI:** 10.1093/geroni/igab046.1280

**Published:** 2021-12-17

**Authors:** Anna Lücke, Cornelia Wrzus, Denis Gerstorf, Ute Kunzmann, Martin Katzorreck, Oliver Schilling

**Affiliations:** 1 Heidelberg University, Heidelberg, Baden-Wurttemberg, Germany; 2 Humboldt University of Berlin, Humboldt University of Berlin, Brandenburg, Germany; 3 Leipzig University, Leipzig, Sachsen, Germany; 4 University of Leipzig, Leipzig, Sachsen, Germany; 5 University of Heidelberg, Heidelberg, Baden-Wurttemberg, Germany

## Abstract

Sufficient sleep is relevant for both momentary cognitive functioning and long-term cognitive developments. However, which factors make people particularly vulnerable to the cognitive consequences of sleep loss remains an open question. Here, we obtained data from 122 young-old (66-69 years) and 35 very old (85-89 years) adults who provided six daily ambulatory assessments of working memory performance and daily sleep over one week, and long-term trajectories in processing speed and working memory performance. Our results add to current knowledge in three ways: First, results from multilevel structural equation models showed both too little and too much daily sleep was associated with poorer working memory in everyday life. Secondly, this association was independent of cognitive aging over the preceding four years. Thirdly, average sleep duration did not predict cognitive changes over the next year. Participants’ age and health as well as emotional functioning are discussed as further influences on the associations.

